# Morphology-Based In-Ovo Sexing of Chick Embryos Utilizing a Low-Cost Imaging Apparatus and Machine Learning

**DOI:** 10.3390/ani15030384

**Published:** 2025-01-29

**Authors:** Daniel Zhang, Leonie Jacobs

**Affiliations:** 1 Maggie L. Walker Governor’s School, Richmond, VA 23220, USA; djzhang1121@gmail.com; 2 School of Animal Sciences, Virginia Tech, Blacksburg, VA 24061, USA

**Keywords:** male chick culling, in-ovo sexing, machine learning, morphology-based analysis, laying hen industry, animal welfare, low-cost technology, non-invasive sexing

## Abstract

The routine culling of male chicks in the laying hen industry raises significant ethical, animal welfare, and sustainability concerns. Current methods to determine chick embryo sex before hatching are costly, time-consuming, and invasive. This study aimed to develop a low-cost, non-invasive solution to predict chick embryo sex before hatching using the morphological features of eggs. A custom imaging apparatus was created using a smartphone and light box, enabling consistent image capture of chicken eggs. Egg length, width, area, eccentricity, and extent were measured, and machine learning models were trained to predict chick embryo sex. The wide neural network model achieved the highest accuracy of 88.9% with a mean accuracy of 81.5%. Comparison of the imaging apparatus to a high-cost industrial 3D scanner demonstrated comparable accuracy in capturing egg morphology. The findings suggest that this method can contribute to the prevention of up to 6.2 billion male chicks from being culled annually by destroying male embryos before they develop the capacity to feel pain. This approach offers a feasible, ethical, and scalable alternative to current practices, with potential for further improvements in accuracy and adaptability to different industry settings.

## Simple Summary

In the laying hen industry, male chicks are typically killed after hatching because they have no economic value. This study developed a low-cost method to predict chick embryo sex before hatching by using a smartphone-based imaging device and machine learning to analyze the shape and size of eggs. The method achieved up to 88.9% accuracy, demonstrating its potential to replace male chick culling by identifying and removing male embryos before they can feel pain. If scaled up, this innovation could greatly reduce the number of male chicks killed each year, improving ethical and sustainable practices in the laying hen industry.

## 1. Introduction

In the laying hen industry, hatcheries incubate and hatch both female and male chicks. However, male chicks have no economic value and are killed shortly after hatch. In the United States (US), approximately 300 million male chicks are culled annually through methods including maceration and carbon dioxide asphyxiation [1]. Across the world, an estimated seven billion male chicks are culled annually [2]. As the human population continues to grow and global food demand increases, the demand for chicken eggs and other sources of animal-based proteins are also expected to grow, potentially further increasing the number of male chicks culled [3].

The practice of mass male chick culling is concerning from ethical, animal welfare, and sustainability perspectives. Ethicists have considered that traditional and humane husbandry in agriculture can allow animals to live a decent life before they are slaughtered, yet the modern approach of culling day-old male chicks places profits over animal lives and raises fundamental questions about the value of animal life [4]. There is concern about male chick culling amongst the public. For instance, 78% of Dutch surveyed citizens found male chick culling unacceptable [5]. Chick culling is an unsustainable practice if culled chicks are discarded. However, they can be used as animal feed, for instance, for snakes [6]. Alternatively, chicks can be raised for meat, yet this is highly inefficient compared to raising broiler chickens. Thus, the resources used to incubate the eggs may ultimately be wasted, while the alternative, raising and slaughtering the males that have little economic value, increases the ecological footprint of the poultry industry [7]. Therefore, research into alternatives to culling is warranted.

Determining embryo sex prior to hatching would avoid the need for live male chick killing. In-ovo methods to predict the sex of a chick embryo would allow eggs with male embryos to be identified and destroyed before the embryos can perceive pain, around embryonic day 13–15 [8]. It would address ethical concerns and improve sustainability. Currently, embryonic sex can be determined by assessing gonad asymmetries with magnetic resonance imaging (MRI), color sexing through visible-near infrared light, and analyzing allantoic fluid extracted from the egg, focusing on biomarkers such as metabolites or DNA [9,10,11,12]. In the European Union, regulations allow these procedures for sex determination, and countries including Germany and France have already fully or partially banned male chick culling, while Italy’s ban goes into effect in 2026 [13]. These methods are 97–99% accurate but can be invasive. Methods involving allantoic fluid require drilling a small hole in the eggshell to acquire a sample for measurement [14]. Opening the eggshell increases the risk of bacterial contamination and can reduce hatchability, which presents a regulatory risk and prevents the adoption of these methods in other countries [15]. Additionally, these methods are often costly, time-consuming (some more than 45 min per egg), and require reagents and sophisticated equipment to extract samples and handle eggs [14]. Hatcheries in the US can process 20,000 eggs an hour; so, any in-ovo sexing technology must keep up with this pace [13]. These current issues with cost, time, and risk of contamination illustrate the value of a low-cost, efficient, and non-invasive method for in-ovo sexing.

A non-invasive analysis of the morphological features of eggs may be used to predict chick embryo sex. While there is no commonly accepted hypothesis for sex-based differences in egg morphology, one possible explanation is the difference in the shape of the egg between male and female embryos. Rounder eggs were thought to contain males, while pointed eggs were thought to contain females [16]. Other studies have also observed differences. The curvature of 103 duck eggs was examined with image-processing and was used to predict duckling sex with 86% accuracy [17]. A machine learning approach was used to predict duckling sex based on morphological features in 503 Philippine Native Duck eggs with 87% accuracy [18]. For chickens, specifically, morphological measurements in combination with machine learning resulted in an 80% accurate prediction of chick embryo sex after testing 47 eggs from a hybrid flock from local backyard poultry farms with unspecified, multiple genetic strains [16]. While these prior studies demonstrate promise for morphological in-ovo sexing of chick embryos, no studies have applied a machine learning approach to commercially used laying hen strains. One paper used Super Nick white layer breeder flock eggs, but the morphological analysis was limited to manually measuring length and width and applying a logistic regression analysis [19]. Furthermore, while patents to use industrial cameras for embryo sexing exist, these studies have relied on image capture through a hand-held camera, instead of a consistent, scalable approach, and these past studies have not validated their imaging method against a highly accurate standard [20].

Therefore, we aimed to use machine learning methods to analyze morphological parameters of chicken eggs and predict chick embryo sex before hatch. A low-cost custom apparatus was developed to enable consistent and stable imaging of eggs. To ensure that the imaging output from the custom apparatus would be accurate, they were compared to images captured by an industrial-grade 3D scanner, a highly accurate method to take morphological measurements, that is costly to purchase (approximately USD 25,000; https://www.artec3d.com/portable-3d-scanners/artec-spider (accessed on 1 July 2023)). We hypothesized that using machine learning to analyze morphological features of eggs would allow for accurate predictions of chick embryo sex. These findings would allow for a low-cost, short-term alternative to male chick culling after hatching, therefore improving ethical treatment, animal welfare, and sustainability in the laying hen industry.

## 2. Materials and Methods

### 2.1. Construction of Imaging Apparatus

**Figure 1:**
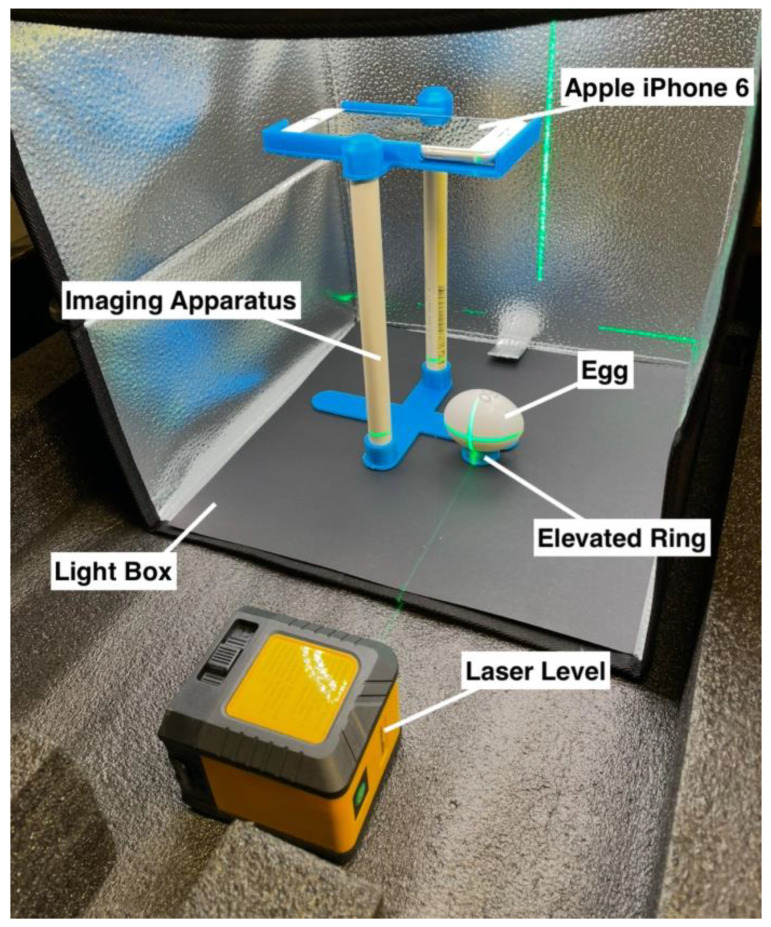
Imaging apparatus with labeled components. This low-cost apparatus was used to standardize image capturing of chicken eggs using a smartphone camera.

To capture consistent images, an apparatus was built that could hold a smartphone at a fixed distance from an egg (Figure 1). The upper platform was designed based on the outline of an iPhone 6 (Apple Inc., Los Altos, CA, USA), leaving an open space for the camera lens. The lower platform had an elevated ring that could secure an egg. Both platforms were 3D printed with PLA plastic and were connected 20 cm apart with two pieces of PVC tubing. The structure was placed inside a light box with reflective interior walls and built-in LED lights to provide consistent lighting and prevent ambient light distortion in the images. The iPhone with the native camera application was used to capture and store images. Aside from the smartphone, the imaging apparatus cost was USD ~60 (USD 30 for light box, USD 20 for laser level, USD 10 for plastic and PVC tubing).

### 2.2. Comparison of Imaging Apparatus to 3D Scanner

The performance of the imaging apparatus was compared to that of a gold-standard professional scanner. Since the smartphone camera produces images that are measured in pixels, features like length and width cannot be directly compared to the scanner measurements, which uses metric units. Because the degree of curvature is intrinsic to the egg shape, regardless of units of measurement, eccentricity [Table 1, Equation (A1)] was calculated with MATLAB R2023b (MathWorks Inc., Natick, MA, USA) and compared across the imaging output of the 10 eggs from both modalities.

To validate the accuracy of the calculations from the smartphone images, 10 non-fertilized eggs were purchased from a supermarket and imaged in both the custom apparatus [Figure 1] and a highly accurate 3D scanner (Artec 3D, Senningerberg, Luxembourg) that is capable of 0.1 mm 3D resolution (Artec Space Spider, 2015). For each egg, its eccentricity [Table 1] was calculated from both the custom apparatus image and the 3D scanner image. The eccentricities were then compared across all 10 eggs through a paired *t*-test.

### 2.3. Chicken Egg Incubation, Imaging, and Sexing

Chicken egg incubation, imaging, and sexing were performed at the Paul B. Siegel Poultry Research Center at Virginia Tech. All procedures were approved by the Virginia Tech Institutional Animal Care and Use Committee (protocol number 23-147). The eggs were obtained by mail and some were damaged in transit. One hundred forty-three fertilized White Leghorn chicken eggs were undamaged, placed in an incubator, and incubated following industry guidelines [15]. This strain is commonly used in the laying hen industry [21]. On day 10 of incubation, the trays of eggs were temporarily removed from the incubator for imaging using the imaging apparatus. Each egg was numbered on the shell with a permanent marker and then placed onto the ring in the imaging apparatus. Using laser levels to ensure the egg was aligned along its horizontal axis to capture the maximum planar projection area, an image was taken with the smartphone. Because the eggs were resting on their sides, the egg centers may have varied by a few millimeters. However, relative to the overall distance from the camera to the egg, any perceivable differences would be minimized. A Bluetooth remote (ASHUTB, Suzhou, China) was used to control the camera shutter without physically interfering with the imaging apparatus or the light box (PULUZ, Shenzhen, China). This process was repeated for the 143 eggs in the study.

On day 20 of incubation, 143 eggs were manually opened, and viable chicks (*n* = 121) were euthanized via cervical dislocation, following the American Veterinary Medical Association’s recommendation. Cervical dislocation was performed by a trained researcher by manually separating the top vertebra from the head using a quick stretching and twisting motion, resulting in a quick death with minimal pain and distress. Then, the trained researcher performed necropsies to observe the chicks’ sexual organs and determine chick sex (male or female).

### 2.4. Chicken Egg Morphology Measurement

**Figure A1:**
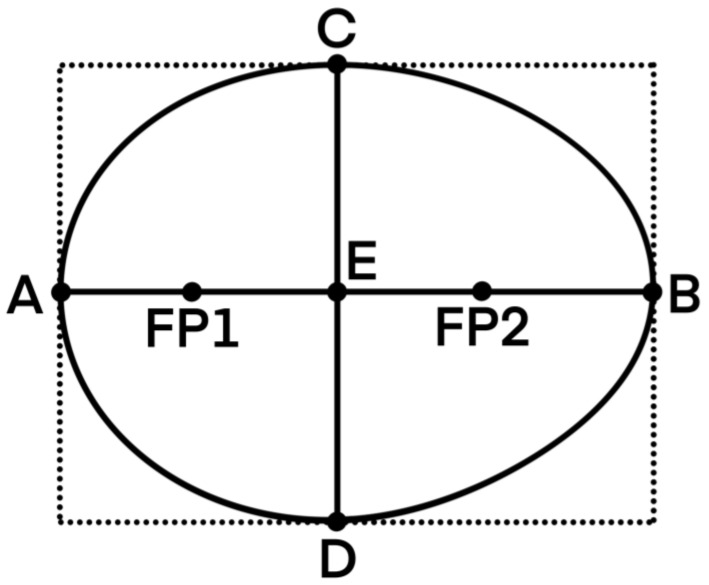
Confusion matrix for *K*-nearest neighbors (medium KNN). A total of 18 of the 121 eggs were reserved to test the predictive accuracy of the model. The diagonal and off-diagonal cells of the matrix show the number of correct and incorrect predictions. The tables to the right and below the matrix display the true positive rates (TPRs), false negative rates (FNRs), positive predictive values (PPVs), and false discovery rates (FDRs). The TPR shows how accurately the model performed in each of the two classes and the FNR is the error rate.

MATLAB version R2023b (MathWorks Inc., Natick, MA, USA) was used to analyze egg morphology. Each black-and-white image was segmented into a binary image with only the outline of the egg against a blank background. The “regionprops” function was used to analyze the morphology of the egg and five features were measured: length, width, area, eccentricity, and extent (Table 1). Eccentricity and extent are measures of roundness; eccentricity is a measure of how a shape deviates from a perfect circle and the extent the area of an object is divided by the area of its bounding rectangle [Appendix A Figure A1]. Of note, all egg images use the same pixel units for dimensions and thus can be directly compared. Experimental records and code used for this analysis are available in a public repository at: https://github.com/danjzg/inovoEgg (accessed on 11 January 2025).

### 2.5. Morphology-Based Machine Learning Analysis

The classification and machine learning toolbox within MATLAB was used to test the accuracy of different models in their ability to predict chick embryo sex based on morphological features. We trained four of the most common and widely used machine learning models, *K*-nearest neighbors (medium KNN), decision tree (boosted trees), support vector machine (cubic SVM), and neural network (wide neural network), with seven-fold cross-validation. In analyzing egg photos, KNN predicts chick sex by looking at similar eggs and choosing the most frequent category (male or female) based on nearby examples [22]. Decision trees split the data into branches using simple rules on features like length and width, and combining many of these small trees improves the overall accuracy of predictions [22]. SVMs work by finding the optimal boundary that best separates male and female classes in the feature space [22]. Wide neural networks use multiple layers of interconnected nodes to capture complex patterns and relationships in the egg features, such as subtle differences in shape, enabling highly accurate sex predictions [23]. A “wide” configuration, characterized by more neurons per layer, captures intricate patterns and complex relationships within the data. Bayesian optimization was conducted for each of the models. The data were randomly (sex-blind) split into 85% (103 eggs) to train the models and 15% (18 eggs) to test the predictive accuracy of the model. A voting mechanism was not used for the testing dataset. Accuracy was expressed as a proportion (%) of the eggs with correctly identified chick embryo sex.

The five morphological features used were ranked in importance using a chi-squared (χ2) feature ranking algorithm in MATLAB R2023b, which is a statistical method used to evaluate the dependency between features and the target variable in classification problems. It quantifies the extent to which the observed frequency distribution of a feature differs from its expected distribution under the assumption of independence; a high χ2 score indicates a strong association between the feature and the target variable, implying that the feature contributes significantly to class discrimination.

## 3. Results

Images taken by the low-cost custom-made imaging apparatus, using a smartphone camera [Figure 1], were tested for accuracy by comparing images of the 10 non-fertilized chicken eggs to images taken with the 3D scanner. A paired t-test indicated that the eccentricity measurements were not significantly different between the imaging apparatus and the 3D scanner (t = 0.142; *p* = 0.89). This indicates that the images from the custom apparatus were sufficiently similar to those from the highly accurate 3D scanner.

Of the 143 fertile chicken eggs incubated, 121 embryos were viable, which were euthanized and had their sex determined. Eleven eggs were infertile and another eleven experienced issues during incubation. The hatched eggs contained 58 female (47.9%) and 63 male chicks (52.1%).

Egg length, width, area, eccentricity, and extent were measured with MATLAB for the morphological assessment [Table 1]. Based on the χ2 ranking algorithm, the width (χ2 = 4.032) and length (χ2 = 2.441) of the eggs were determined to be the most important predictive features for embryo sex, followed by area (χ2 = 0.857), extent (χ2 = 0.743), and eccentricity (χ2 = 0.298) [Table 1]. Each machine learning algorithm was trained and tested three times, each time randomly splitting the dataset into training and testing sets. The wide neural network produced the most accurate results with an accuracy high of 88.9% and a range of 11.1% [Table 2]. The next most accurate algorithm was the boosted trees model, with a high of 83.8% and a range of 28.2% [Table 2]. The cubic SVM and medium KNN models produced lower accuracies, with highs of 72.2% and 55.6%, and ranges of 22.2% and 11.2%, respectively [Table 2].

**Figure 2:**
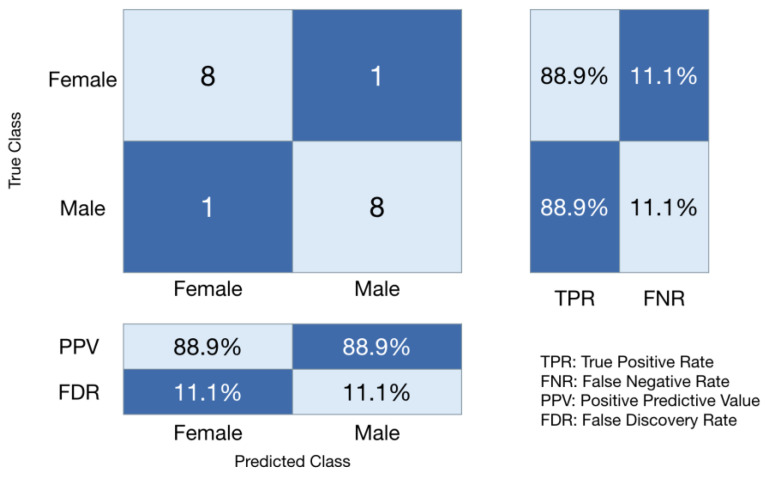
Confusion matrix for neural network (wide neural network). A total of 18 of the 121 eggs were reserved to test the predictive accuracy of the model. The diagonal and off-diagonal cells of the matrix show the number of correct and incorrect predictions. The tables to the right and below the matrix display the true positive rates (TPRs), false negative rates (FNRs), positive predictive values (PPVs), and false discovery rates (FDRs). The TPR shows how accurately the model performed in each of the two classes (88.9%) and the FNR is the error rate (11.1%).

**Figure 3:**
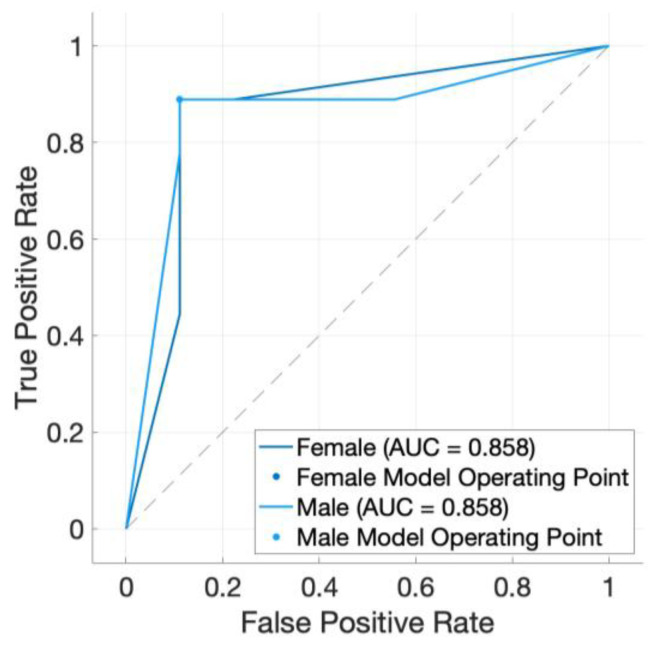
Receiver Operating Characteristic (ROC) curve for neural network (wide neural network). The Model Operating Point, in the top-left corner, represents the ideal compromise between sensitivity and specificity. The area under the curve (AUC) value represents the overall quality of the model, where values closer to one indicate a strong model performance.

**Figure A2:**
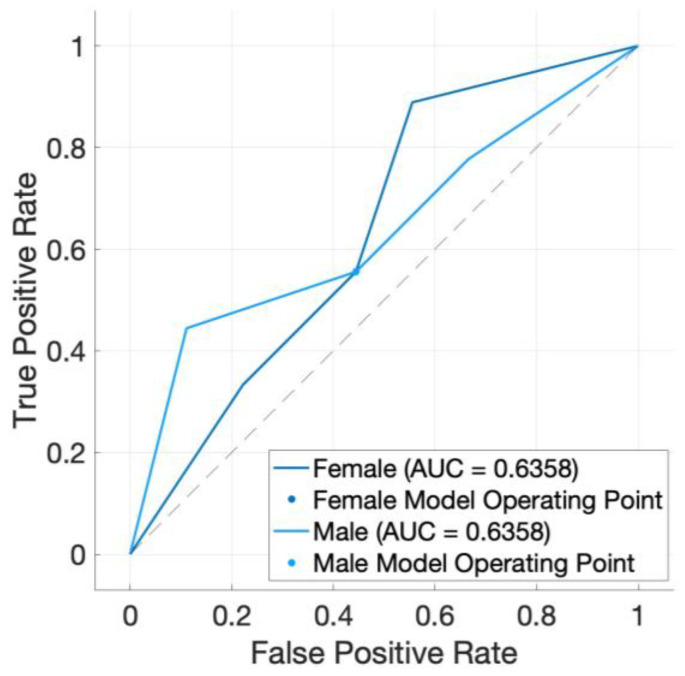
Receiver Operating Characteristic (ROC) curve for *K*-nearest neighbors (medium KNN). The Model Operating Point, in the top-left corner, represents the ideal compromise between sensitivity and specificity. The area under the curve (AUC) value represents the overall quality of the model, where values closer to one indicate a strong model performance.

**Figure A3:**
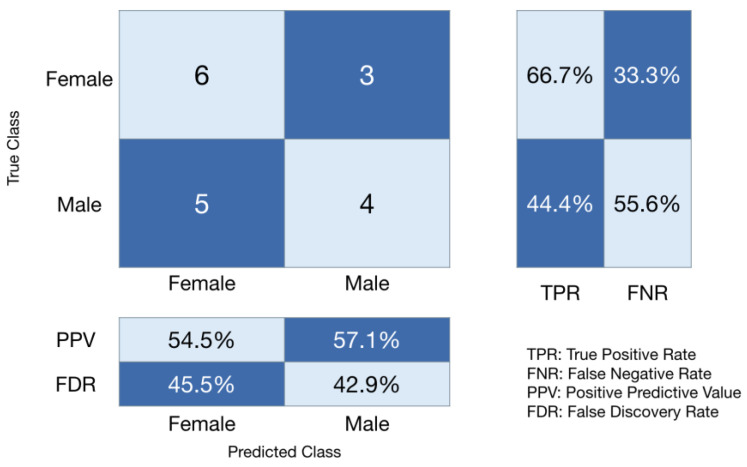
Confusion matrix for decision tree (boosted trees). A total of 18 of the 121 eggs were reserved to test the predictive accuracy of the model. The diagonal and off-diagonal cells of the matrix show the number of correct and incorrect predictions. The tables to the right and below the matrix display the true positive rates (TPRs), false negative rates (FNRs), positive predictive values (PPVs), and false discovery rates (FDRs). The TPR shows how accurately the model performed in each of the two classes and the FNR is the error rate. Of note, this model did not perform equally between the two sexes, leading to variations between the TPRs, FNRs, PPVs, and FDRs of each sex.

**Figure A4:**
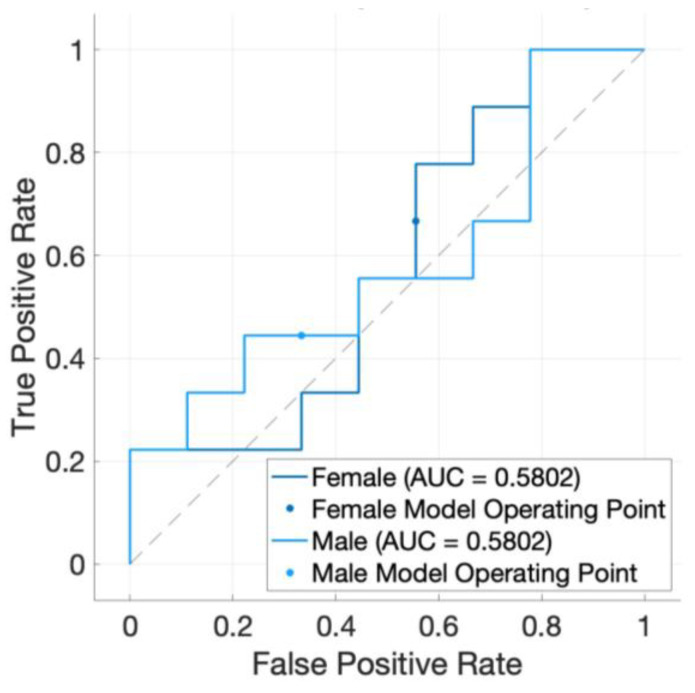
Receiver Operating Characteristic (ROC) curve for decision tree (boosted trees). The Model Operating Point, in the top-left corner, represents the ideal compromise between sensitivity and specificity. The area under the curve (AUC) value represents the overall quality of the model, where values closer to one indicate a strong model performance.

**Figure A5:**
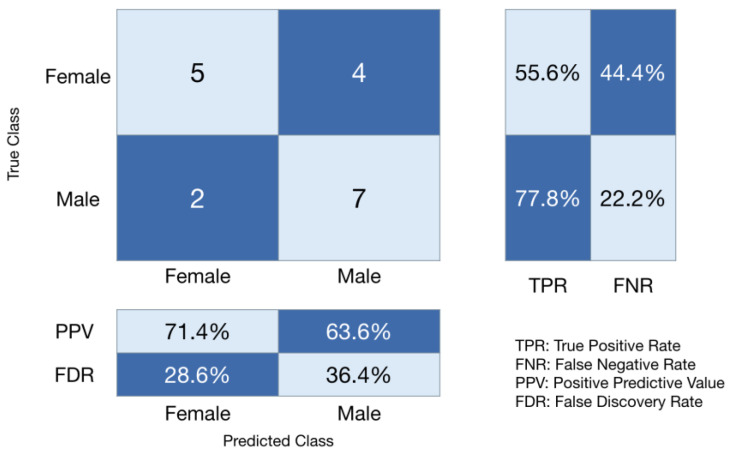
Confusion matrix for support vector machine (cubic SVM). A total of 18 of the 121 eggs were reserved to test the predictive accuracy of the model. The diagonal and off-diagonal cells of the matrix show the number of correct and incorrect predictions. The tables to the right and below the matrix display the true positive rates (TPRs), false negative rates (FNRs), positive predictive values (PPVs), and false discovery rates (FDRs). The TPR shows how accurately the model performed in each of the two classes and the FNR is the error rate.

**Figure A6:**
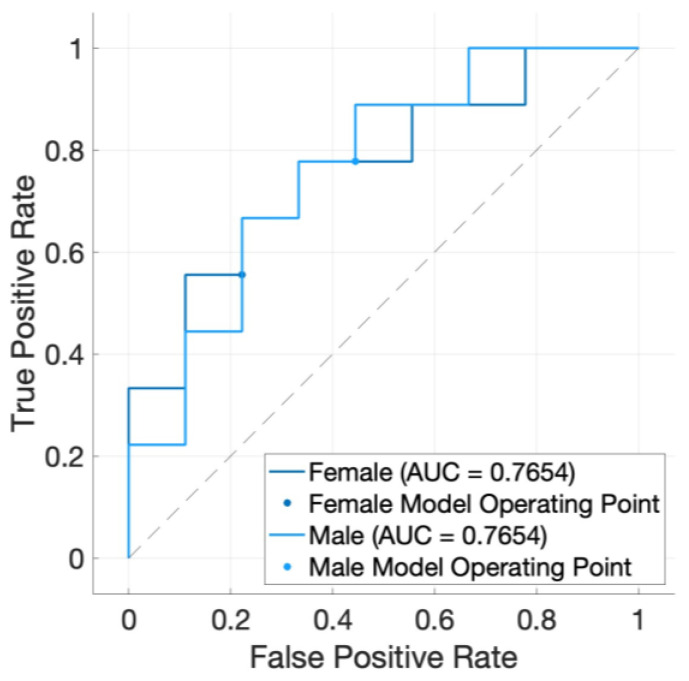
Receiver Operating Characteristic (ROC) curve for support vector machine (cubic SVM). The Model Operating Point, in the top-left corner, represents the ideal compromise between sensitivity and specificity. The area under the curve (AUC) value represents the overall quality of the model, where values closer to one indicate a strong model performance.

**Figure A7:**
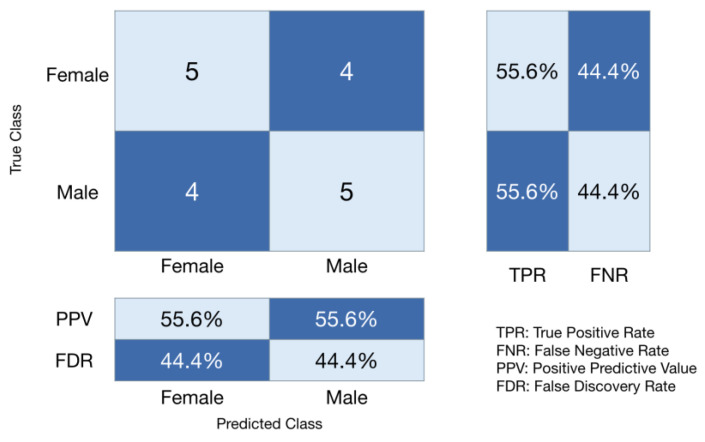
Diagram of an egg with defined variables for eccentricity and extent calculations.

A confusion matrix [Figure 2] was created based on the wide neural network’s test results from trial 2 [Table 2] and shows the quality of the algorithm predictions. In this study, the positive predictive value and false discovery rate mirror the function of the true positive rate and false negative rate due to a coincidentally symmetrical testing set and model performance. A receiver operating characteristic (ROC) curve was also created based on the wide neural network’s performance from trial 2 [Figure 3]. Since the wide neural network performed identically between the two sexes, the model operating points and area under the ROC curve are the same. The value of 0.858 for the area under the ROC curve demonstrates strong model performance. Our findings reveal that the wide neural network had the capability to predict both sexes with high and equal accuracy. For comparison purposes, confusion matrices and ROC curves were created for the other three models in trial 2 [Appendix A Figure A2, Figure A3, Figure A4,Figure A5, Figure A6 and Figure A7.

## 4. Discussion

The aim of this study was to accurately predict chicken embryo sex based on egg morphology using a low-cost apparatus. Based on data from 121 eggs, we found that the wide neural network was able to produce the highest predictive accuracies, up to 88.9% with a mean accuracy of 81.5%. These are within the range reported in previous studies (80–87%) [16,17,18], supporting the hypothesis that a machine-learning-based analysis of morphological measurements of chicken eggs is a reliable method of non-invasive in-ovo chick sexing.

We observed variability in performance across the three trials. These variations in predictive accuracy across trials are primarily due to the random splitting of testing and training data, which is a necessary and inherent step to avoid overfitting of the partitioning of the dataset during cross-validation. Random splits of data into training and testing subsets can lead to differences in the underlying data distributions available for model training. This is particularly impactful for algorithms like KNN, which rely on the proximity of data points in feature space [22]. Variations in the composition of training and testing sets can affect the algorithm’s ability to generalize, leading to variable results across trials.

The neural network’s ability to adapt to complex, nonlinear relationships and its capacity to learn hierarchical feature representations likely enabled it to outperform the other algorithms. Neural networks excel at capturing nonlinear patterns in data due to their layered structure and activation functions [23]. Unlike decision trees or KNN, which have fixed mechanisms for decision-making, the neural network’s iterative learning approach allows for continuous improvement in accuracy during training. While a neural network requires more computational resources and careful tuning compared to simpler models, these advantages make neural networks particularly effective in scenarios where the data are sufficiently complex and abundant. The observed superior performance in this case suggests that the dataset’s characteristics aligned well with the strengths of the neural network architecture.

In this study, only non-linear machine learning algorithms were tested. Linear (linear discriminant) and partial linear (binary generalized linear model logistic regression) algorithms were considered but yielded low accuracies in initial iterations.

The image results from our custom imaging apparatus and smartphone were compared to an industrial-grade 3D scanner. We found that the smartphone can produce a statistically similar output compared to the industrial scanner, which demonstrates that low-cost solutions are able to produce high-quality images for morphological assessment. The implementation of this method, when scaled to a commercial setting, has the potential to contribute to the prevention of up to 88.9% of hatched chicks from being culled, which would be 6.2 billion male chicks annually around the world out of the 7 billion currently being culled. Rather, the eggs could be destroyed at embryonic day 10, before chick embryos can experience pain, which is estimated to start around embryonic day 13 [8]. It is likely that earlier imaging, for instance, prior to incubation, is possible to predict embryonic sex; however, the effect of developmental stage was not included in the scope of this study. We recommend the inclusion of embryonic age as a factor in future research. The 11.1% misclassification rate of female embryos as male would lead to needless destruction of up to 778 million female embryos. Thus, further improvements to accuracy and precision are warranted to reach a consistent 98–99% accuracy rate.

Related studies have focused on other methods to classify chick embryo sex. Some researchers are using genetic engineering to introduce detectable labels in male embryo genomes [24]. However, there are concerns about the risks and regulations around genetically modified organisms. Assessing gonad asymmetries with MRI, color sexing through visible-near infrared light, and analyzing metabolites or DNA in allantoic fluid extracted from the egg have already been implemented commercially [9,10,11,12]. However, each method has limitations, with the former two being underdeveloped or being too narrowly effective and the latter being an invasive technique [25]. Fluorescence and Raman spectroscopy has been a promising minimally invasive approach to in-ovo sexing, and researchers have developed ways to perform the procedure without breaking the inner shell membrane, though a window cut into the outer shell membrane is still required [26]. Nonetheless, with continued concerns about contamination and impact on hatching rates, the need for additional non-invasive in-ovo sexing methods remains.

Since White Leghorn chickens are not the only strain of chickens used in the laying hen industry, this study could be expanded by testing eggs from other strains. Furthermore, future trials could have larger sample sizes, which would reduce the impact of possible sampling errors. Synthetic augmentation of the dataset has proven to be a reliable technique and would greatly increase the sample size without the need to sample more eggs [27]. Perhaps a larger sample size and inclusion of other egg factors, such as egg weight, could improve the model and further increase accuracy rates. Increased accuracy rates would eliminate false-culling concerns. While the mean accuracy is 81.5%, an average of 19.5% of eggs were incorrectly identified, which would mean that some males would still be hatched and some eggs with females would mistakenly be destroyed. The design of the imaging apparatus could also be improved to be adaptable to more real-world situations and the scale of the laying hen industry. For instance, the camera could be suspended over a conveyor belt, imaging eggs as they pass by, or the camera could be adjusted to image an entire incubating tray at once. Also, in small hatching volume settings such as on small farms, a custom smartphone application with the morphological algorithm onboard could enable small-scale farmers to make individual chick embryo sex predictions.

This study developed and validated a low-cost solution to consistently image chicken eggs for morphological analysis and in-ovo sex prediction, with a mean accuracy of 81.5%. The results indicate the feasibility of preventing or reducing male chick culling in the laying hen industry, which would be a major improvement in terms of the ethical treatment of animals, animal welfare for male chicks, and industry sustainability.

## 5. Conclusions

This study demonstrated a promising and scalable method for in-ovo chick embryo sexing using a low-cost smartphone-based imaging apparatus combined with machine learning analysis of egg morphology. By achieving a mean accuracy of 81.5% and a peak accuracy of 88.9%, this non-invasive technique offers a viable alternative to current invasive or resource-intensive methods. The innovation addresses pressing ethical concerns and sustainability challenges in the laying hen industry, with the potential to prevent billions of male chicks from being culled annually by identifying and removing male embryos before they can experience pain. Future work should aim to improve the accuracy of this approach by incorporating additional egg features, testing on diverse chicken strains and developmental stages, and refining the apparatus for commercial scalability.

## Figures and Tables

**Table 1:** Classification features used for morphological analysis of eggs.

Morphological Feature	Description
Length	Maximum pixel distance between any two opposite points of the outline of an egg.
Width	Minimum pixel distance between any two opposite points of the outline of an egg.
Area	Number of pixels within the outline of an egg.
Eccentricity	Ratio of the pixel distance between the foci of the ellipse and its length [Equation (A1)].
Extent	Ratio of pixels inside the outline of an egg and the total number of pixels in a bounding box around the outline of the egg [Equation (A2)].

**Table 2:** Accuracy of model performances predicting chick embryo sex based on egg morphology.

MATLAB Algorithms	Trial 1	Trial 2	Trial 3	Mean
*K*-nearest neighbors (medium KNN)	47.6%	55.6%	44.4%	49.2%
Decision tree (boosted trees)	83.8%	55.6%	72.2%	70.5%
Support vector machine (cubic SVM)	50.0%	66.7%	72.2%	63.0%
Neural network (wide neural network)	77.8%	88.9%	77.8%	81.5%

## Data Availability

The raw data supporting the conclusions of this article will be made available by the authors on request.

## Appendix A

(A1)
$$\mathrm{Eccentricity}=\left|\frac{\mathrm{FP1FP2}}{\mathrm{AB}}\right|$$

(A2)
$$\mathrm{Extent}=\left|\frac{\mathrm{circumference}\;\mathrm{of}\;\mathrm{egg}}{\mathrm{perimeter}\;\mathrm{of}\;\mathrm{box}\;\left(\mathrm{dotted}\right)\;\mathrm{bounding}\;\mathrm{the}\;\mathrm{egg}}\right|$$

Equations (A1) and (A2). Equations used to calculate eccentricity and extent values from egg images.
